# AAV-based gene therapies for neovascular AMD

**DOI:** 10.1038/s41434-026-00595-4

**Published:** 2026-02-13

**Authors:** Tae Hee Kim, Chan You Kwon, Jae Yoon Song, Hee Chan Yoo

**Affiliations:** 1 College of Pharmacy, Chung-Ang University, Seoul, Republic of Korea; 2 Organoid Medical Center, Chung-Ang University, Seoul, Republic of Korea

**Keywords:** Genetic vectors, Diseases

## Abstract

Neovascular age-related macular degeneration (nAMD) is a major cause of irreversible vision loss in the elderly, driven by choroidal neovascularization and dysregulated vascular endothelial growth factor (VEGF) signaling. While anti-VEGF injections have transformed management, their frequent administration imposes a substantial burden on patients and limits adherence. Adeno-associated virus (AAV)-based gene therapy offers sustained intraocular delivery of anti-angiogenic agents with a single treatment, potentially overcoming these limitations. This review summarizes the rationale for AAV use in ocular gene therapy, compares major delivery routes, and highlights leading clinical candidates, including RGX-314, ADVM-022, 4D-150, and NG101. Advances in vector engineering, promoter optimization, and immune modulation are discussed alongside key challenges such as preexisting immunity and inflammation. Future directions include next-generation capsids, combination regimens, and precision patient selection. Collectively, these developments position AAV-based gene therapy as a promising strategy to redefine the therapeutic landscape of nAMD.

## Introduction

Age-related macular degeneration (AMD) is a progressive retinal disease and a major cause of central vision impairment in individuals over the age of 50 [1]. Globally, it is estimated that approximately 8.69% of individuals aged 45–85 years are affected by AMD. Clinically, AMD is classified into two main types: dry (non-neovascular) and wet (neovascular) [2]. Dry AMD, accounting for ~85–90% of all AMD cases, is characterized by drusen accumulation, retinal pigment epithelium (RPE) atrophy, and gradual photoreceptor degeneration [3]. In contrast, neovascular AMD (nAMD), though less prevalent, is responsible for the majority of severe and rapid vision loss associated with the disease [4, 5].

**Fig. 1:**
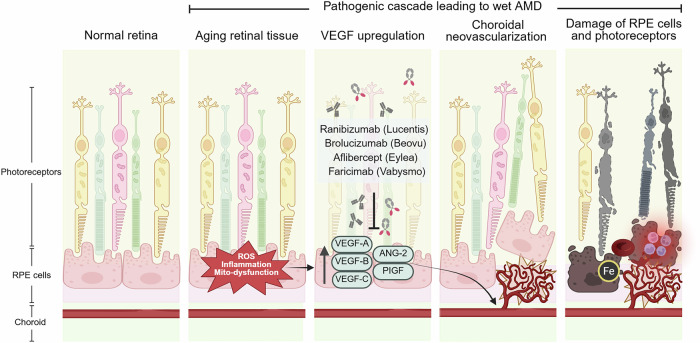
Pathogenesis of nAMD and therapeutic intervention. In a healthy eye, the retina’s layers, including light-sensing photoreceptors and the supportive RPE, are nourished by the underlying choroidal vasculature. With aging, factors such as oxidative stress, inflammation, and mitochondrial dysfunction increase VEGF expression. Excessive VEGF signaling drives CNV, characterized by abnormal vessel growth from the choroid into the retina. These leaky vessels cause hemorrhage and release toxic substances such as iron (Fe) and plasma proteins, leading to RPE and photoreceptor degeneration and ultimately vision loss. Abnormal vessels also induce inflammation and immune activation, further damaging the retina. Current therapies, including anti-VEGF agents (ranibizumab [Lucentis], aflibercept [Eylea], brolucizumab [Beovu], faricimab [Vabysmo]), suppress VEGF activity to reduce vascular leakage and slow disease progression, thereby preserving vision.

This subtype is characterized by the pathological development of choroidal neovascularization (CNV), wherein abnormal blood vessels originating from the choroid penetrate through Bruch’s membrane into the subretinal (SR) space [6, 7]. These newly formed vessels are leaky and structurally immature, thereby increasing vascular permeability, macular edema, SR hemorrhage, and subsequent damage to the photoreceptors and the RPE [8]. The primary molecular driver of this neovascularization is vascular endothelial growth factor (VEGF), which is upregulated in response to oxidative stress, hypoxia, and retinal inflammation in the aging eye [4] (Fig. 1).

Current standard therapy for nAMD involves repeated intravitreal (IVT) injections of anti-VEGF agents, such as ranibizumab, brolucizumab, aflibercept (targeting VEGF-A, VEGF-B, and placental growth factor (PIGF)) and faricimab (targeting VEGF-A and angiopoietin-2 (ANG-2)) [9, 10]. These biologics act by neutralizing VEGF-A (and additional targets as noted) and inhibiting its interaction with VEGF receptors on endothelial cells, thereby suppressing neovascular growth and vascular leakage [11]. While these treatments have significantly improved visual outcomes, they necessitate frequent administration, often monthly or bimonthly, to maintain efficacy [12]. This treatment burden affects both patients and healthcare systems, resulting in poor adherence, incomplete disease suppression, and recurrent disease activity.

In light of these limitations, gene therapy has emerged as a safe and promising alternative, capable of providing sustained therapeutic benefits through a single intervention [13]. Among various gene delivery platforms, adeno-associated virus (AAV) vectors have demonstrated exceptional promise due to their ability to transduce non-dividing retinal cells [1, 14], mediate long-term gene expression, and exhibit minimal immunogenicity in ocular tissues [15]. AAV-mediated expression of anti-VEGF proteins could obviate the need for frequent injections, thereby enhancing treatment adherence and clinical outcomes [16].

This review aims to provide a comprehensive overview of recent advances in AAV-based gene therapy approaches specifically targeting nAMD. We discuss the underlying disease mechanisms, current limitations of anti-VEGF therapies, progress in AAV vector engineering, and the status of ongoing clinical trials. In doing so, we highlight how AAV gene therapy could potentially reshape the therapeutic landscape for patients suffering from this vision-threatening condition.

## Neovascular AMD (wet AMD)

nAMD is a vision-threatening subtype of macular degeneration characterized by the pathological growth of blood vessels from the choroidal vasculature into the SR space, a process termed CNV [17]. These neovessels are structurally immature, with increased permeability and fragility [18]. The resulting accumulation of SR or intraretinal fluid disrupts the retinal architecture and compromises the structural and functional integrity of both photoreceptors and the underlying RPE, ultimately leading to central vision loss (Fig. 1) [17, 18].

The molecular pathogenesis of nAMD is driven primarily by overexpression of angiogenic factors, with VEGF-A being the most critical [19, 20]. VEGF-A stimulates endothelial cell proliferation, migration, and new vessel formation through activation of VEGF receptors, particularly VEGFR-2. Additional isoforms such as VEGF-B and PlGF also contribute to the neovascular cascade, albeit to a lesser extent [19]. These factors are upregulated in response to oxidative stress, mitochondrial dysfunction, and chronic inflammation—all hallmarks of aging retinal tissue [21–23]. The cumulative effect of these molecular insults leads to a pro-angiogenic microenvironment that promotes CNV formation [21] (Fig. 1).

Accurate diagnosis of nAMD relies heavily on advanced retinal imaging modalities. Optical coherence tomography (OCT) allows for cross-sectional visualization of retinal layers and is critical for detecting SR and intraretinal fluid, pigment epithelial detachments, and retinal thickening [24, 25]. Fluorescein angiography (FA) remains a valuable tool for assessing the presence, extent, and activity of CNV by visualizing leakage patterns [25]. More recently, OCT angiography (OCTA) has emerged as a non-invasive method to map the neovascular networks without the need for dye injection [26, 27].

Upon clinical diagnosis of nAMD, patients are typically initiated on anti-VEGF therapy following either a fixed dosing regimen (e.g., monthly or bimonthly injections), a pro re nata (PRN, “as-needed”) approach based on disease activity, or a treat-and-extend (T&E) protocol, in which dosing intervals are progressively lengthened if disease remains quiescent [28]. These regimens aim to balance therapeutic efficacy with the burden of repeated IVT injections.

Currently approved biologics include four major agents, each with distinct molecular mechanisms. Ranibizumab is a monoclonal antibody fragment that specifically binds VEGF-A, thereby preventing receptor activation and subsequent neovascularization and vascular leakage. Brolucizumab, a single-chain antibody fragment, also targets VEGF-A but is structurally smaller, which allows for higher molar dosing per injection and potentially longer durability of VEGF suppression [11, 29]. Aflibercept is a recombinant fusion protein combining the ligand-binding domains of VEGFR-1 and VEGFR-2 with the Fc portion of IgG1, functioning as a high-affinity “VEGF trap” that neutralizes VEGF-A, VEGF-B, and PIGF [30]. Faricimab is a bispecific antibody that targets both VEGF-A and ANG-2, thereby inhibiting VEGF-driven angiogenesis while simultaneously stabilizing vascular integrity through modulation of the Tie2 signaling pathway [30, 31].

Although anti-VEGF therapies have demonstrated substantial efficacy, they still present areas where therapeutic strategies could be refined. These agents require a regimen of repeated injections, typically every 4 to 8 weeks, to sustain therapeutic benefit [32]. While they have transformed the clinical management of nAMD, real-world data suggest that long-term visual outcomes are often suboptimal due to under-treatment and patient dropout [33].

Many patients experience incomplete response or develop tachyphylaxis (a gradual reduction in treatment effectiveness despite continued administration), necessitating a switch to alternative therapeutic agents [34–36]. Moreover, the burden of frequent clinic visits and invasive procedures contributes to poor adherence and treatment fatigue [37]. Approximately one-third of patients fail to achieve significant visual improvement even after regular therapy [38]. These challenges underscore the need for novel treatment modalities that offer durable efficacy with reduced treatment burden, an unmet need that gene therapy approaches aim to address.

## AAV as a vector for ocular gene therapy

**Fig. 2:**
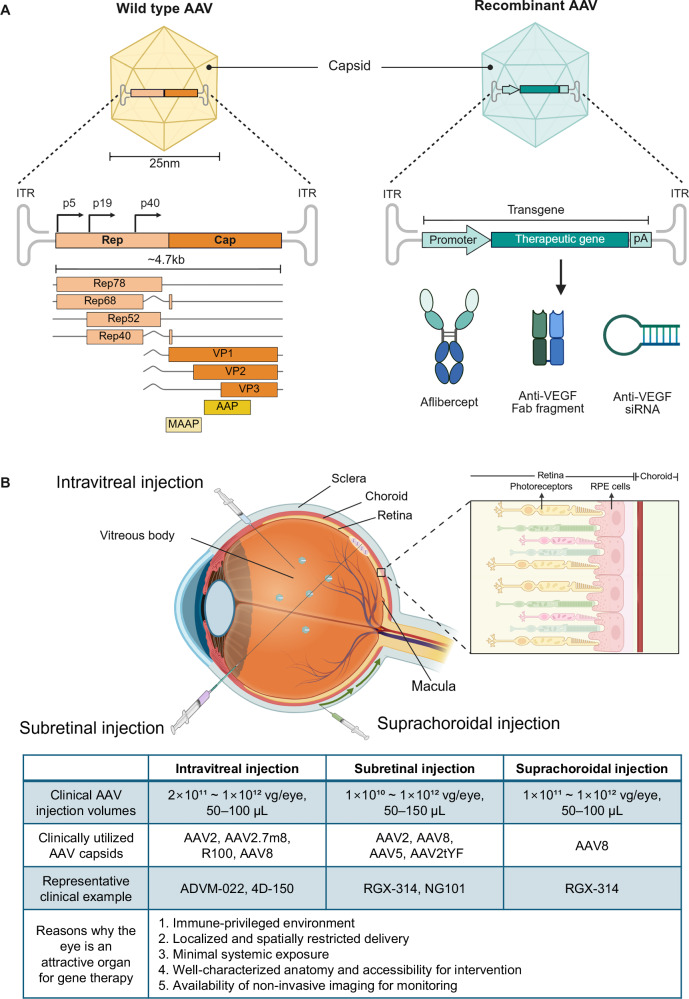
AAV vector structures and ocular delivery routes. **A** Structural comparison of wild-type and rAAV particles. Left, wild-type AAV encapsidates the native genome containing *Rep* and *Cap* genes between ITRs. Right, rAAV encapsidates a therapeutic expression cassette in place of the Rep–Cap region, while replication and capsid functions are provided in trans during production. Both assemble into ~25 nm icosahedral capsids. In rAAV, the transgene cassette (promoter–coding sequence–pA, flanked by ITRs) can drive expression of therapeutic proteins such as aflibercept or anti-VEGF Fab fragments, or introduce synergistic modulators such as anti-VEGF siRNAs. **B** Overview of ocular gene therapy routes. Three main methods for injecting gene therapy vectors into the eye are intravitreal injection, SR injection and SC injection. Each route is chosen to target specific retinal layers. Intravitreal Injection: A needle injects the vector into the vitreous body (the gel in the center of the eye). This method allows the vector to disperse over a wide area, reaching cells in the inner retina. Subretinal Injection: This more invasive method injects the vector into the space between the retina and the RPE. This allows for a highly localized and concentrated delivery, ideal for targeting photoreceptors and RPE cells. Suprachoroidal Injection: The vector is injected into the space between the choroid and the sclera. This targets cells in the outer retina and choroid while avoiding the vitreous body.

AAV is a small (~25 nm), non-enveloped virus of the *Parvoviridae* family containing a ~4.7 kb single-stranded DNA (ssDNA) genome [15]. The capsid is assembled from 60 subunits comprising three structural viral proteins, VP1, VP2, and VP3, at an approximate molar ratio of 1:1:10 [39]. The AAV genome is flanked by inverted terminal repeats (ITRs) and encodes two major open reading frames (ORFs), *rep* and *cap* [39, 40]. The *rep* gene gives rise to four non-structural proteins, Rep78, Rep68, Rep52, and Rep40, that are essential for viral genome replication, transcriptional regulation, and packaging [40]. The *cap* gene encodes not only the capsid proteins VP1–3, but also two accessory proteins: the assembly-activating protein (AAP) and the membrane-associated accessory protein (MAAP), which are involved in capsid assembly and viral egress [40] (Fig. 2A).

Conventional viral vector-based gene therapies have primarily utilized γ-retroviruses and lentiviruses [41–43]; however, these vectors integrate transgenes into the host genome, which poses a potential risk of tumorigenesis [44, 45]. In addition, as most of them employ the vesicular stomatitis virus glycoprotein (VSV-G) envelope, they can be rapidly inactivated by the complement system in human serum upon in vivo administration, thereby eliciting innate and adaptive immune responses [46]. Consequently, to minimize immune reactions, ex vivo strategies involving the transplantation of pre-transduced cells are predominantly employed. However, because the eye and central nervous system (CNS) are immune-privileged sites, localized in vivo application of lentiviral vectors is feasible, although vector leakage into the bloodstream can increase immunogenicity [46, 47]. In fact, RetinoStat, an equine infectious anemia virus (EIAV)-based lentiviral vector expressing endostatin and angiostatin for the treatment of nAMD (NCT01301443, NCT01678872), was administered locally via SR injection in clinical studies for this reason [42, 48, 49].

In contrast, AAVs are replication-deficient and require co-infection with a helper virus for productive replication, rendering them inherently safe for therapeutic applications [50]. In addition, AAV genomes rarely integrate into the host genome, thereby minimizing the risk of insertional mutagenesis, and instead persist as stable episomal forms that enable long-term transgene expression [51]. AAV vectors have demonstrated a strong safety profile, minimal pathogenicity, and the ability to transduce both dividing and non-dividing cells, making them particularly well-suited for use in the retina, where many target cells, such as photoreceptors and RPE, are post-mitotic [14, 52]. Numerous naturally occurring AAV serotypes (e.g., AAV2, AAV5, AAV8, AAV9) and engineered variants offer diverse tropisms and tissue-specific targeting capabilities, providing a versatile toolkit for retinal gene delivery [8, 15, 16, 52, 53]. Furthermore, AAV vectors enable fine-tuned cell-type targeting through capsid engineering and promoter optimization, allowing selective gene expression in photoreceptors, RPE, or retinal ganglion cells (RGCs). Their well-established current Good Manufacturing Practice (cGMP) manufacturing platforms and multiple regulatory approvals (e.g., Luxturna, Zolgensma) further underscore their proven clinical safety, scalability, and translational maturity compared to other viral vector systems.

The immune-privileged status of the eye, supported by the blood–retina barrier that limits systemic exposure, further enhances the suitability of AAV vectors for ocular gene therapy [54, 55]. Moreover, the enclosed anatomical compartments of the eye, such as the vitreous cavity and SR space, permit spatially restricted gene delivery, thereby reducing off-target distribution and enabling therapeutic transgene expression at comparatively lower vector doses [56]. These unique features make the eye an ideal organ for evaluating and advancing in vivo gene therapy (Fig. 2B).

Several delivery routes have been developed to administer AAV vectors to the retina, each with distinct advantages and limitations. SR injection involves surgical delivery of the vector between the RPE and photoreceptor layers, resulting in high transduction efficiency of these cell types [57, 58]. However, this method is inherently invasive, requiring pars plana vitrectomy, a surgical procedure in which the vitreous gel inside the eye is removed to access the retina, and specialized surgical expertise, and may lead to serious complications such as vitreous hemorrhage and retinal detachment when performed under nonstandard conditions [59, 60]. Moreover, transduction is typically restricted to localized areas near the injection site, limiting its applicability for treating pan-retinal diseases [61] (Fig. 2B).

IVT injection represents a minimally invasive and clinically feasible approach for retinal gene delivery, enabling broad transgene distribution across the inner retina, particularly targeting RGCs. However, its transduction efficiency is often constrained by anatomical barriers such as the inner limiting membrane (ILM) and tends to elicit stronger systemic immune responses compared with SR [62–64]. Moreover, because IVT administration inherently relies on passive diffusion of AAV particles within the vitreous, achieving therapeutically relevant transduction generally necessitates the use of high vector doses. This requirement, in turn, increases the likelihood of dose-dependent ocular inflammation and systemic immune activation, which remain key safety considerations in the clinical application of this route [61].

Suprachoroidal (SC) injection provides minimally invasive access to the potential space between the sclera and choroid, enabling widespread transduction of the outer retina, including the RPE, without disrupting the blood–retinal barrier, serving as a less invasive alternative to SR delivery [65, 66]. Consequently, SC administration has garnered increasing attention as a promising strategy for therapeutic indications in which selective and efficient targeting of the RPE layer is particularly desirable [67–69] (Fig. 2B).

## AAV capsid engineering and vector optimization

**Fig. 3:**
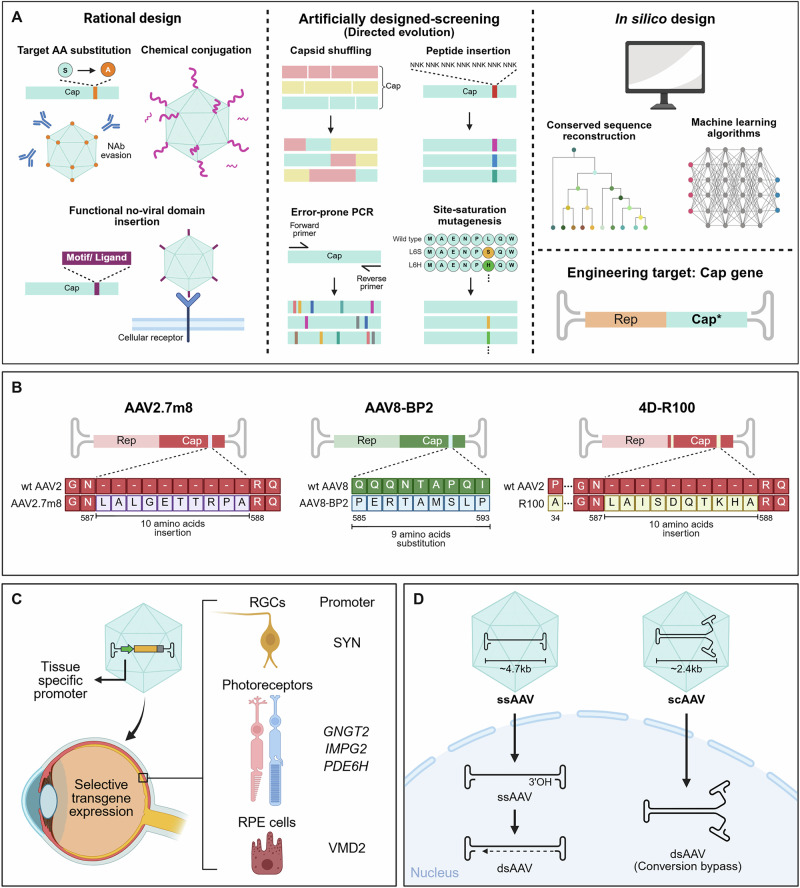
Strategies for AAV capsid engineering and applications in retinal gene therapy. **A** Schematic of major strategies for AAV capsid engineering. Rational design uses targeted substitutions, chemical conjugation, or motif/ligand insertion. Artificially designed screening (directed evolution) applies random mutagenesis and library-based iterative selection, using approaches such as shuffling, error-prone PCR, peptide insertion, or saturation mutagenesis. In silico design leverages conserved-sequence reconstruction and machine learning to predict optimized variants. **B** Representative engineered AAV variants and their defining modifications. AAV2.7m8 harbors a 10–amino acid insertion at VP1 residue 588. AAV8-BP2 harbors a 9–residue substitution at residues 585–593 of AAV8. 4D-R100 harbors a 10–residue insertion at VP1 residue 588 together with an N-terminal VP1 substitution (Pro34Ala). **C** Gene therapy for the retina aims to deliver therapeutic genes to specific cell types. Different promoters are used to preferentially drive gene expression in target cells. SYN promoter targets RGCs. *GNGT2*, *IMPG2*, and *PDE6H* promoters target photoreceptors. VMD2 promoter targets RPE cells. **D** Comparison of ssAAV and scAAV genome configurations and processing. ssAAV packages a ~4.7 kb single-stranded genome requiring second-strand synthesis in the nucleus to generate transcriptionally active dsDNA. In contrast, scAAV carries a ~2.4 kb self-complementary genome that folds into a double-stranded form upon uncoating, bypassing second-strand synthesis and enabling faster transgene expression.

The efficiency and specificity of AAV-mediated gene delivery, determined by the capsid structure that governs tropism, immunogenicity, and intracellular trafficking, have been markedly improved through recent advances in capsid engineering using rational design, directed evolution, and in silico approaches [70–72]. Rational design capitalizes on structural and functional knowledge of the AAV capsid to optimize delivery efficiency, refine tissue tropism, and attenuate immunogenicity. Representative strategies encompass targeted amino acid substitutions; incorporation of functional non-viral peptides such as cell-penetrating peptides (CPPs) or integrin-binding ligands; and chemical conjugation methods such as polyethylene glycol (PEG) or N-acetylgalactosamine (GalNAc) coupling [39, 40, 73]. Directed evolution generates genetic diversity through random and unbiased mutagenesis (e.g., peptide insertion, error-prone PCR, DNA shuffling, and site-saturation mutagenesis) and employs iterative diversification–selection cycles to isolate capsid variants with desirable properties [39, 74], such as enhanced penetration through the ILM [75] or improved transduction of specific retinal cell types [76]. In contrast, in silico design leverages conserved-sequence reconstruction and machine learning algorithms trained on large-scale mutagenesis data to rationally predict and generate novel AAV capsids with optimized properties such as enhanced packaging, tissue tropism, and immune evasion (Fig. 3A) [71, 77].

One of the central goals of capsid engineering is to enhance retinal cell tropism while minimizing immune recognition [78]. These advancements are particularly important for overcoming anatomical barriers such as the vitreous and the ILM, which hinder efficient gene delivery via non-invasive routes. To address these challenges, engineered capsids—including AAV2.7m8, AAV8-BP2, and 4D-R100—have demonstrated markedly improved transduction efficiency in RGCs, photoreceptors, and RPE cells following IVT injection [75, 79, 80]. AAV2.7m8 was developed through in vivo screening of AAV2 using a seven–amino acid insertion library, which converged on a variant containing a ten–amino acid insertion (LALGETTRPA) at residue R588 of VP1 (loop IV, hypervariable region VIII) (Fig. 3B). This insertion disrupts the key arginine residues R585 and R588 within loop IV responsible for heparan sulfate proteoglycan (HSPG) binding, thereby enhancing ILM penetration and enabling broad retinal transduction following IVT administration [79, 81, 82]. In both mice and non-human primates (NHPs), rAAV2.7m8 efficiently targets RGCs, interneurons, photoreceptors, and RPE, providing a less invasive alternative to SR delivery. In relevant disease models, 7m8-mediated delivery of retinoschisin 1 (RS1) in *Rs1h*-/- mice restored synaptic organization and rescued visual function, while AAV2.7m8-RPE65 administration in the *rd12* mouse model of Leber’s congenital amaurosis (LCA) achieved robust RPE65 expression and a corresponding rescue of vision [81].

AAV8-BP2 was obtained through a directed selection strategy using an NNK saturation mutagenesis library targeting residues 585–593 of the AAV8 capsid protein. This loop represents the most protruding region of the AAV8 capsid and plays a key role in receptor interaction and cell entry. Among the variants, BP2 (PERTAMSLP) retained capsid integrity while markedly enhancing ON-bipolar cell transduction in mice after IVT injection [83] (Fig. 3B). However, in NHPs, AAV8-BP2 mainly transduced RGCs and the optic nerve, likely due to the thicker retinal barriers in primates [80].

4D-R100 was similarly engineered from an AAV2 backbone through in vivo screening in NHPs, incorporating a 10–amino acid insertion (LAISDQTKHA) at residue 588 and a VP1 point mutation at position 34 (Pro→Ala). These modifications reduce HSPG affinity and promote alternative glycan interactions (notably sialic acid), facilitating enhanced ILM penetration and efficient retinal transduction across RPE, photoreceptors, and RGCs from macula to periphery following IVT administration. Furthermore, IVT delivery of an R100 vector carrying an anti-VEGF transgene effectively suppressed grade IV angiogenic lesions in a primate model of laser-induced CNV [75, 84] (Fig. 3B). Although their immunogenicity profiles differ, these capsids offer meaningful progress toward enabling safe and effective IVT gene therapy.

Beyond capsid design, transcriptional regulation of the transgene is another area of active optimization. Utilization of tissue-specific promoters enables spatially restricted and potentially inducible transgene expression, thereby enhancing therapeutic precision and minimizing off-target effects and transcription-associated toxicity [85] (Fig. 3C). For instance, the vitelliform macular dystrophy-2 (VMD2; *BEST1*, bestrophin-1) promoter can drive strong and selective transgene expression in RPE cells, making it a widely used promoter for RPE-targeted applications [86]. G protein subunit gamma transducin 2 (*GNGT2*), interphotoreceptor matrix proteoglycan 2 (*IMPG2*) and phosphodiesterase 6H (*PDE6H*) promoters have been developed to achieve high levels of transgene expression in photoreceptors (rods and/or cones), and their use may help reduce immune activation and improve safety [87]. For RGCs, the synapsin (SYN) promoter has demonstrated high specificity and robust expression, making it a suitable candidate for delivering axon regeneration–associated genes directly to RGCs [88] (Fig. 3C).

Additionally, self-complementary AAV (scAAV) vectors, which bypass the rate-limiting step of second-strand synthesis required by ssAAV, accelerate transgene expression onset and enhance expression levels across multiple tissues, including the retina [89]. This feature is particularly advantageous for treating rapidly progressing diseases such as nAMD. However, this design reduces the packaging capacity to ~2.4 kb, roughly half that of ssAAV, posing challenges for the delivery of larger genes or complex regulatory elements [90] (Fig. 3D).

Designing the therapeutic transgene in conjunction with an efficient expression cassette is a critical determinant of the efficacy and specificity of AAV-mediated gene therapy. Within the limited packaging capacity of AAV (~4.7 kb), several strategies can be employed to maximize expression and functionality. These include codon optimization to enhance translational efficiency [91], incorporation of secretion signals to ensure proper extracellular localization of therapeutic proteins [92], and rational design of minimal yet fully functional expression cassettes [93]. The integration of multi-target constructs, such as bispecific antibodies [94] or dual-gene cassettes [68], allows simultaneous modulation of multiple disease pathways and can be particularly beneficial in complex conditions like nAMD.

## Current AAV-based therapeutic strategies and clinical trials for nAMD

A growing number of AAV-based gene therapy candidates for nAMD are in preclinical or clinical development, with most focusing on sustained intraocular expression of anti-angiogenic proteins to reduce or replace frequent IVT injections of recombinant anti-VEGF agents (Table 1). These approaches typically employ distinct AAV serotypes, delivery routes, and transgene constructs, aiming for durable therapeutic protein expression after a single administration. Notable examples in ongoing clinical trials include RGX-314, ADVM-022, 4D-150, and NG101, which illustrate diverse combinations of AAV serotypes, administration routes, and transgene designs.

**Table 1:** Summary of AAV-based gene therapy candidates in clinical development for AMD.

Candidate	Sponsor	Capsid	Route of administration	Transgene	Stage	NCT number	Others
RGX-314	REGENXBIO	AAV8	SR	a soluble anti-VEGF protein	Phase I/II	NCT03066258	Phase III ongoing; both SR and SC delivery evaluated; Dose: 3 × 10⁹ –2.5 × 10¹¹ vg/eye
		AAV8	SR	an anti-VEGF Fab fragment	Phase II	NCT03999801	
		AAV8	SR	an anti-VEGF Fab fragment	Phase II	NCT04832724	
		AAV8	SC	an anti-VEGF Fab fragment	Phase II	NCT04514653	
		AAV8	SR	an anti-VEGF Fab fragment	Phase II/III	NCT04704921	
		AAV8	SR	an anti-VEGF Fab fragment	Phase III	NCT05407636	
ADVM-022	Adverum Biotechnologies	AAV2.7m8	IVT	aflibercept	Phase I	NCT03748784	Phase III ongoing; engineered capsid AAV2.7m8 used; Dose: 6 × 10¹⁰ –6 × 10¹¹ vg/eye
		AAV2.7m8	IVT	aflibercept	Phase II	NCT05536973	
		AAV2.7m8	IVT	aflibercept	Phase III	NCT06856577	
4D-150	4D Molecular Therapeutics	4D-R100	IVT	Codon-optimized gene encoding aflibercept and VEGF-C-targeting miRNA	Phase I/II	NCT05197270	Synthetic R100 capsid optimized for IVT; Dose: 6 × 10⁹ –3 × 10¹⁰ vg/eye
NG101	Elisigen(Neuracle Genetics)	AAV8	SR	aflibercept	Phase I/II	NCT05984927	Choroid–retina-targeted synthetic promoter (CAT311) used [116]; Dose: 1 × 10⁹ – 8 × 10⁹ vg/eye (very low dose)
LX102	Innostellar Biotherapeutics	AAV2	SR	a VEGF-trap protein	Phase I	NCT06198413	Dose: 2 × 10¹⁰–1.25 × 10¹¹ vg
		AAV2	SR	a VEGF-trap protein	Phase II	NCT06196840	
AAV2-sFLT01	Genzyme, a Sanofi Company	AAV2	IVT	sFLT01	Phase I	NCT01024998	Dose: 2 × 10⁸–2 × 10¹⁰ vg/eye
rAAV.sFlt-1	Lions Eye Institute, Perth, Western Australia	AAV2	SR	sFLT01	Phase I/II	NCT01494805	Dose: 2 × 10⁸–2 × 10¹⁰ vg/eye
AAVCAGsCD59	Janssen Research & Development	AAV2	IVT	sCD59	Phase I	NCT03585556	sCD59 (complement inhibitor gene therapy evaluated for dry AMD); Cohorts: young participants (1–25 years); Dose: 3.56 × 10¹¹ –1.07 × 10¹² vg/eye
KH631	Chengdu Origen Biotechnology	AAV8	SR	an anti-VEGF protein	Phase I	NCT05657301	Well tolerated at 6.0 × 10¹⁰ vg/eye; Dose: 1.0 × 10¹⁰ –2.5 × 10¹¹ vg/eye
		AAV8	SR	an anti-VEGF protein	Phase I/II	NCT05672121	
SKG0106	Youxin Chen	N/A	IVT	an anti-VEGF protein	Phase I	NCT06213038	N/A
	Skyline Therapeutics	N/A	IVT	an anti-VEGF protein	Phase I/II	NCT05986864	N/A
EXG202	Hangzhou Jiayin Biotech Ltd	N/A	IVT	a fusion protein of ABD–VEGFR	Early Phase I	NCT06888492	N/A
EXG102-031	Hangzhou Jiayin Biotech	N/A	SR	a fusion protein of ABD–VEGFR	Phase I/II	NCT06183814	
		N/A	SR	a fusion protein of ABD–VEGFR	Phase I	NCT05903794	
		N/A	SR	a fusion protein of ABD–VEGFR	Phase I	NCT06817343	
HG202	HuidaGene Therapeutics	N/A	SR	CRISPR/Cas13 RNA-editing system targeting VEGFA	Early Phase I	NCT06031727	N/A
		N/A	SR	CRISPR/Cas13 RNA-editing system targeting VEGFA	Phase I	NCT06623279	
KH658	Chengdu Origen Biotechnology	N/A	SC	an anti-VEGF protein	Phase I/II	NCT06458595	N/A

RGX-314 was jointly developed by REGENXBIO and AbbVie.

*SR* subretinal, *SC* suprachoroidal, *IVT* intravitreal, *VEGF* vascular endothelial growth factor, *sFLT01* soluble fms-like tyrosine kinase-1, *sCD59* soluble CD59, Fab fragment antigen-binding, SAE serious adverse event, *CAT311* cis-acting transcriptional element 311, *miRNA* microRNA, *ABD* angiopoietin-binding domain, *CRISPR* clustered regularly interspaced short palindromic repeats, *Cas* CRISPR-associated protein, *N/A* not available, *AAV* adeno-associated virus.

NCT numbers refer to ClinicalTrials.gov identifiers.

### RGX-314 (Regenxbio/AbbVie)

RGX-314 is one of the most advanced candidates, employing an AAV8 vector to deliver a transgene encoding an anti-VEGF antibody fragment analogous to ranibizumab [95–97]. It is being evaluated via SR injection, which enables direct RPE transduction, and SC injection using a proprietary microneedle system [98–100]. These clinical outcomes were further supported in subsequent trials [96, 98, 100]. In the Phase I/IIa SR dose-escalation trial, higher-dose cohorts (≥6 × 10¹⁰ vg/eye) maintained protein expression for up to 2 years, with stable best-corrected visual acuity (BCVA) and central retinal thickness (CRT), and required 59–85% fewer anti-VEGF injections compared with baseline, while lower-dose groups showed minimal benefit [98]. In the Phase II AAVIATE trial, SC administration of RGX-314 showed encouraging clinical outcomes. Early interim data from Cohorts 1 and 2 (2.5 × 10¹¹ and 5.0 × 10¹¹ vg/eye) showed no drug-related serious ocular adverse events, with mild intraocular inflammation occurring in approximately 23% of patients and resolving with topical corticosteroids. Visual and anatomical outcomes remained stable, with up to 40% of treated eyes remaining injection-free for 6 months. Subsequent dose expansion to 1.0 × 10¹² vg/eye (dose level 3) maintained a consistent safety profile and yielded an ≈80% reduction in annualized injection frequency, with 50% of eyes remaining injection-free at 6 months [101]. Additionally, in patients previously treated unilaterally, SR administration of RGX-314 (1.3 × 10¹¹ vg/eye) in the fellow eye reduced the annualized anti-VEGF injection frequency by 92.8% (from 8.8 to 0.7 injections/year), with 60% remaining injection-free over one year. Collectively, BCVA and CRT remained stable, and no drug-related serious adverse events or intraocular inflammation were observed, supporting the safety and durability of bilateral AAV-mediated gene therapy for nAMD [102]. However, clinical outcomes from the ongoing Phase IIb/III and Phase III trials remain to be reported.

### ADVM-022 (Adverum Biotechnologies)

ADVM-022 (ixoberogene soroparvovec; Ixo-vec) employs an engineered AAV2.7m8 capsid for IVT delivery of aflibercept to suppress multiple angiogenic pathways [96, 103]. Early clinical data from the OPTIC trial indicated sustained therapeutic protein levels and anatomical improvements, though some concerns were raised regarding intraocular inflammation, necessitating further optimization of vector dosing [104]. In this Phase I study, participants were assigned to four cohorts differing in vector dose and prophylactic corticosteroid regimen—receiving either a low (2 × 10¹¹ vg/eye) or high (6 × 10¹¹ vg/eye) dose of Ixo-vec with oral prednisone or topical difluprednate prophylaxis. A single IVT administration achieved sustained aflibercept expression, reducing annual injection frequency by 85–99% in the first year, with most patients remaining injection-free. Two-year follow-up confirmed maintenance of visual and anatomical benefits without additional anti-VEGF therapy [105, 106]. The most common adverse event was mild-to-moderate anterior segment inflammation, generally responsive to topical corticosteroids, underscoring the need for steroid co-administration and vector dose or formulation optimization [79, 80]. Collectively, long-term follow-up from the OPTIC study demonstrated durable efficacy of low-dose Ixo-vec (2 × 10¹¹ vg/eye), with maintained visual and anatomical benefits, up to 86% reduction in annual anti-VEGF injection frequency over four years, and an overall favorable safety profile responsive to topical corticosteroids [107]. In the Phase II LUNA trial, a single IVT administration of Ixo-vec (6 × 10¹⁰ or 2 × 10¹¹ vg/eye) maintained stable BCVA and CST through 52 weeks while reducing annualized anti-VEGF injection frequency by 88–92%, with 54–69% of patients remaining injection-free without additional anti-VEGF injections. Both doses were well tolerated, and inflammation was effectively controlled with topical corticosteroids, with no new onset beyond week 30. Based on these findings, the 6 × 10¹⁰ vg/eye dose combined with topical-only prophylaxis was selected for the pivotal Phase III program [107, 108].

### 4D-150 (4D molecular therapeutics)

4D-150 utilizes a fully synthetic R100 capsid, derived via directed evolution in NHPs, optimized for IVT administration [109, 110]. Its dual-transgene cassette encodes a codon-optimized aflibercept and an miRNA targeting VEGF-C [109–111]. This multi-pathway approach aims for greater efficacy than VEGF-A monotherapy. Preclinical studies demonstrated broad retinal transduction and robust expression in human retinal cells and NHPs [75, 84]. In the Phase I/II PRISM trial, patients with nAMD received a single IVT administration across three dose cohorts (6 × 10⁹, 1 × 10¹⁰, and 3 × 10¹⁰ vg/eye). The vector was well tolerated at all dose levels, with no dose-limiting toxicities or treatment-related serious ocular adverse events. In the highest-dose cohort (3 × 10¹⁰ vg/eye), the annualized anti-VEGF injection frequency decreased by 96.7% (from 10.8 to 0.36 injections/year), and 80% of eyes required no supplemental aflibercept injections during 12–40 weeks of follow-up [112]. In the randomized Phase II dose-expansion stage, participants received a single IVT injection of 4D-150 (1 × 10¹⁰ or 3 × 10¹⁰ vg/eye) or aflibercept 2 mg every 8 weeks. No ≥ Grade 1 inflammation, retinal vasculitis, or hypotony was reported during 12–36 weeks of follow-up, and both visual and anatomic outcomes were comparable to those of standard aflibercept therapy [113]. In the Phase IIb population-expansion cohort (3 × 10¹⁰ vg/eye), interim analysis demonstrated sustained clinical activity and favorable tolerability. Over 32–52 weeks of follow-up, 73% of eyes remained injection-free, 93% required ≤1 injection, and BCVA and CST were maintained or improved. No treatment-related serious adverse events, intraocular inflammation, or vascular complications were observed [114].

### NG101 (Elisigen, Neuracle Genetics)

NG101 is an AAV8-based SR-delivered aflibercept gene therapy [115, 116]. Aflibercept expression is driven by a synthetic CAT311 promoter derived from the cytomegalovirus immediate-early enhancer/chicken β-actin (CAG) promoter arrangement. Preclinical optimization enabled robust and sustained expression at relatively low vector doses. In NHP models with laser-induced CNV, NG101 markedly reduced lesion size and leakage. In cynomolgus monkeys administered 3 × 10⁹ or 6 × 10⁹ vg/eye via SR injection, therapeutic efficacy was comparable to that achieved with 2 mg aflibercept (Eylea) administered via IVT injection, and aflibercept expression persisted for up to 1 year. These findings suggest that NG101 may provide efficacy comparable to Eylea in patients with nAMD while reducing treatment burden through sustained intraocular aflibercept expression. Moreover, unlike other clinical candidates, NG101 achieved effective VEGF suppression at lower vector doses in NHPs, potentially mitigating AAV-related toxicity associated with high-dose vector administration [116]. A Phase I/IIa open-label trial (NCT05984927) is currently recruiting patients with neovascular AMD previously treated with anti-VEGF therapy, evaluating safety, tolerability, and preliminary efficacy across three dose cohorts (1 × 10⁹, 3 × 10⁹, and 8 × 10⁹ vg/eye), representing substantially lower vector doses than those used in other ongoing trials [115].

Collectively, these programs demonstrate that AAV gene therapy can provide durable anti-VEGF expression for up to 24 months post-injection, significantly reducing treatment burden [98, 103, 105, 106, 112–116]. However, long-term expression raises concerns about overexpression toxicity [117], and re-administration remains challenging due to neutralizing antibodies (NAbs) [118]. Potential solutions—such as transient immunosuppression or orthogonal capsids—are under active investigation [119, 120]. Patient-centric considerations also influence clinical adoption: IVT and SC delivery are more acceptable and scalable than SR surgery [66], but achieving consistent efficacy across patient populations remains a priority as these therapies move toward approval and real-world use.

Emerging strategies extend beyond VEGF-A blockade, including dual inhibition of VEGF and ANG-2 to enhance vascular stabilization [121], as well as AAV-mediated RNA interference (RNAi) or clustered regularly interspaced short palindromic repeats (CRISPR)–CRISPR-associated (Cas) systems for precise, gene-specific regulation [67, 122, 123]. Short hairpin RNA (shRNA) or microRNA (miRNA) targeting *VEGF* transcripts could serve as non-protein-based alternatives, while CRISPR-based tools could modulate *VEGF* expression at the genomic level. For example, AAV-delivered Ago2-dependent short hairpin RNA (shRNA) constructs placed in primary microRNA (miRNA) scaffolds (miR-agshRNA) have shown potent and highly specific *VEGFA* silencing by eliminating the passenger strand, thereby minimizing off-target activity and Dicer-related cytotoxicity inherent to conventional shRNA systems. Validated in a porcine laser-induced CNV model following SR AAV2.8 delivery, this platform demonstrated significant suppression of neovascular lesions [67]. In a subsequent study, dual VEGF-targeting miR-agshRNA units were co-delivered with the anti-angiogenic protein pigment epithelium–derived factor (PEDF) via SR injection in C57BL/6JRj mice, showing additive inhibition of CNV formation while preserving retinal integrity [122], further underscoring the therapeutic versatility and safety of RNAi-based AAV systems for nAMD. However, clinically, most ongoing nAMD gene therapy programs still focus on gene supplementation strategies that express anti-VEGF biologics such as aflibercept, soluble Flt-1 (sFLT-1), and antibody fragments, whereas RNAi- or CRISPR-based approaches remain far less represented. RNAi-based candidates are currently being evaluated in combination with aflibercept, while CRISPR-based research has shifted from the traditional SpCas9-mediated genomic knockout strategy toward Cas13-driven RNA-level knock-down approaches (Table1). This reflects persistent concerns that permanent DNA editing may lead to unpredictable off-target mutations or other adverse effects following direct administration in humans [124–126]. Although these modalities have not yet reached clinical translation, their continued refinement and encouraging experimental outcomes suggest that RNA- and CRISPR-based AAV therapeutics may ultimately complement existing anti-VEGF gene supplementation paradigms for nAMD.

## Challenges and considerations

**Fig. 4:**
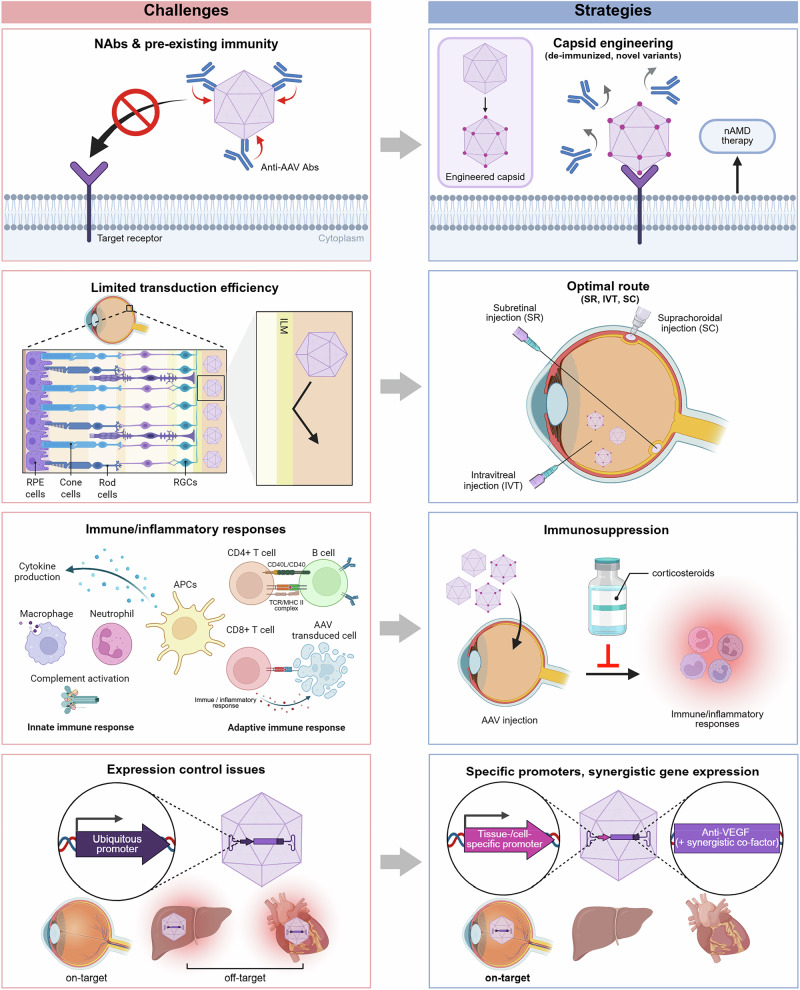
Challenges and potential strategies in AAV-based gene therapy for neovascular AMD. Key challenges include (1) preexisting NAbs that limit patient eligibility and re-dosing, (2) barriers to efficient target cell transduction requiring optimized delivery routes (SR, IVT, SC), (3) innate and adaptive immune responses that compromise safety and efficacy, and (4) restricted control of transgene expression due to limited vector packaging capacity. Potential strategies include engineered capsids with reduced seroprevalence, optimization of delivery routes, transient immunosuppression, combination gene therapy (e.g., anti-VEGF with ANG-2 or PEDF), and compact, cell-specific promoters and enhancers to achieve precise and durable expression.

Despite the considerable promise of AAV-based gene therapy for nAMD, several key challenges must be addressed to ensure widespread clinical success. One of the most significant limitations is the presence of preexisting NAbs against AAV capsids in the human population [64, 127, 128]. These antibodies, which may arise from natural exposure to wild-type AAVs, can neutralize the vector before it reaches target tissues, reducing transduction efficiency and potentially rendering patients ineligible for treatment [129]. Furthermore, a single exposure to an AAV vector can prime the immune system, complicating future re-dosing efforts and raising safety concerns [130] (Fig. 4).

The route of administration entails specific advantages and limitations. SR delivery enables direct targeting of the RPE and photoreceptors, resulting in high transduction efficiency and spatially confined transgene expression [104, 131]. However, this approach necessitates a surgical procedure, which limits its routine applicability and may be contraindicated in patients with advanced retinal changes, where formation of an SR bleb is not feasible [58]. In contrast, IVT administration is minimally invasive and readily integrated into routine clinical practice [131], although its efficacy may be limited by anatomical barriers such as the ILM and by humoral immune factors within the vitreous [104]. SC delivery represents a potential intermediate approach, offering a balance between procedural invasiveness and target tissue accessibility, yet requiring further clinical validation to establish its safety and efficacy [17, 104, 132] (Fig. 4).

Another important consideration is the risk of toxicity from transgene overexpression, especially when high vector doses are needed to reach therapeutic levels [133]. Although AAV vectors typically support long-term and well-tolerated protein production, excessive or ectopic expression can disrupt retinal homeostasis or trigger immune-mediated inflammation [134, 135]. This risk is heightened by the limited packaging capacity of AAV vectors, which constrains the use of complex regulatory elements that could otherwise fine-tune expression [135]. Achieving precise cell-type targeting also remains difficult, as existing promoters and capsids may not provide adequate specificity across the heterogeneous cell populations of the retina, potentially reducing efficacy or causing off-target effects [136] (Fig. 4).

Beyond these biological and technical hurdles, the manufacturing and scalability of AAV vectors present additional translational challenges. The production of high-quality AAV at clinical-grade and commercial scales remains labor-intensive and costly, with issues such as batch-to-batch variability, low yields, and vector purity that must be resolved to enable large-scale deployment [137–139]. Moreover, the high development and manufacturing costs are reflected in the projected pricing of gene therapies, potentially limiting patient access and reimbursement. Regulatory agencies are also developing more comprehensive frameworks to evaluate the safety, durability, and long-term follow-up of ocular gene therapies, further adding complexity to the approval and commercialization process [140, 141]. Addressing these manufacturing, economic, and regulatory barriers will be essential to translate recent scientific and preclinical advances into safe, scalable, and broadly accessible AAV-based treatments for nAMD.

These multifaceted challenges have spurred ongoing innovation in vector design, delivery strategies, and translational methodologies, paving the way for next-generation AAV-based therapies.

## Future directions

Future progress in AAV-based gene therapy for nAMD will be shaped by innovations in vector design, immune modulation, clinical trial strategy, and translational development. In response to these challenges, one of the most promising areas of research is the development of next-generation capsids with enhanced cell- and tissue-specificity, transduction efficiency, and immune evasion. Synthetic AAV capsids, designed through directed evolution and machine learning-guided in silico screening [142, 143], are being tailored for specific delivery routes such as IVT or SC injection. These capsids are optimized for improved retinal penetration and cell-type targeting, and can be engineered to evade preexisting NAbs, a major barrier to eligibility and redosing (Fig. 4).

Combination therapy, such as transient systemic or local immunosuppression, represents a promising approach to enhance efficacy and expand patient eligibility in AAV-based gene therapy. This strategy can attenuate anti-capsid immune responses and has shown potential to enable transduction in NAb-positive subjects, and its use is increasingly explored in clinical settings [144, 145]. In addition to immune modulation, co-expression of molecules that modulate angiogenic signaling has been proposed as a complementary strategy. For example, ANG-2 inhibition can synergize with VEGF blockade by stabilizing vascular integrity and reducing neovascular lesion progression, thereby serving as a rational co-target in combination gene therapy approaches [146]. Beyond ANG-2, several candidates have been investigated for potential benefit when expressed alongside aflibercept. PEDF has demonstrated potent anti-angiogenic and neuroprotective properties in preclinical models, offering additive effects when combined with VEGF suppression [147, 148]. Similarly, endostatin and angiostatin have shown efficacy in reducing CNV formation and vascular leakage, supporting their relevance as adjunct therapeutic genes [42]. Incorporating such co-factors into AAV-based constructs may enhance treatment durability, broaden therapeutic impact, and mitigate resistance mechanisms often observed with anti-VEGF monotherapy.

A critical consideration in future clinical development is the establishment of NAb threshold cutoffs to refine patient selection and increase trial success rates [149]. The implementation of standardized assays and validated NAb titer thresholds, exemplified by AAV6 enrollment assays that, through analyses of clinical trial data, demonstrate a correlation between preexisting NAb levels and transgene expression, enables more precise stratification of patients into likely responders and non-responders, thereby optimizing trial enrollment and minimizing variability in clinical outcomes [150].

Bridging the gap between preclinical validation and human application remains a major translational hurdle [151]. Although NHPs provide the closest anatomical and functional resemblance to the human eye, their use is often avoided due to ethical concerns and economic burden. Consequently, alternative animal models such as mice, rats, rabbits, dogs, and pigs are frequently employed, despite notable interspecies differences in retinal architecture, photoreceptor composition, and the absence of a macula, which may lead to divergent outcomes when translated to humans [152].

To address interspecies differences, patient-derived retinal organoids and post-mortem explants are gaining attention as promising next-generation models for future preclinical evaluation. Recent advances in stem cell biology have enabled the generation of human induced pluripotent stem cell (hiPSC)-derived retinal organoids, providing a patient-specific and ethically acceptable alternative to embryonic stem cell–based models. hiPSCs retain the donor’s genetic background, allowing disease modeling under authentic genetic contexts [153, 154]. Three-dimensional (3D) retinal organoids derived from hiPSCs recapitulate the layered architecture and cellular diversity of the human retina, offering a reproducible and scalable platform for disease modeling, drug screening, and evaluation of AAV transduction efficiency and cell-type specificity [153]. For example, when AAV-mediated delivery of h*CRB1* or h*CRB2* was performed in retinal organoids differentiated from hiPSCs carrying pathogenic *CRB1* mutations, both Müller glia and photoreceptor phenotypes were partially restored at the morphological and transcriptional levels [155]. These findings underscore the potential of human organoids to complement animal studies and bridge species gaps in assessing AAV efficacy. However, retinal organoids still exhibit structural and physiological limitations, including the absence of a fully organized RPE layer, the lack of a macular region, and an inability to recapitulate the complex ocular microenvironment. Notably, the current organoid systems do not incorporate vascular barriers such as the blood–retinal and blood–brain barriers, thereby limiting their utility for assessing AAV vector penetration, systemic delivery dynamics, and immune interactions. Moreover, incomplete synaptic connectivity hampers functional assays such as electrophysiological recordings, restricting the capacity to evaluate photoreceptor responses and network-level restoration [156].

In parallel, ex vivo human retinal explant models derived from donor tissues have emerged as valuable tools for AAV ocular gene therapy [157]. Retinal explants preserve the authentic tissue polarity and cellular composition of the human retina, facilitating direct assessment of vector tropism and transduction efficiency under near-physiological conditions [158]. Application of an AAV2 peptide display library to human retinal explants enabled functional screening of novel capsids, leading to the identification of AAV2-M4, which demonstrated superior transduction in human Müller glia compared with AAV2.7m8, while showing limited activity in mouse retina. This highlights the translational relevance of explant-based screening for identifying human-specific AAV variants [159]. Nevertheless, human explant models face several challenges, including inter-donor variability in genetic and environmental backgrounds, limited reproducibility, potential axonal stress induced by retinal detachment, and the inability to fully replicate in vivo biochemical and biomechanical environments. In addition, ethical and administrative constraints—such as donor consent and institutional review board (IRB) approval—add complexity to their routine use [158].

Although patient-derived retinal organoids and human retinal explants are still limited in their ability to replicate the complex structural and functional organization of the human retina, continued refinement through advanced bioengineering approaches is expected to greatly enhance their translational value. With further optimization, these systems may develop into robust preclinical platforms that more accurately predict human outcomes and accelerate the development of safer and more effective AAV-based ocular gene therapies.

## Conclusion

AAV-based gene therapy represents a transformative shift in the treatment of nAMD, offering the possibility of durable disease control with a single intervention. By enabling long-term intraocular expression of anti-angiogenic agents, these approaches could significantly reduce the burden of repeated injections while preserving or improving vision. While immune challenges, targeting precision, and delivery optimization remain to be addressed, continued innovation in vector design and combination strategies is rapidly closing these gaps. As these therapies transition from trials to clinical practice, they may not only redefine AMD management but also serve as a model for gene therapy in other chronic, vision-threatening diseases.
